# Physical Exercise After Fragility Fractures: A Systematic Review and Meta-Analysis of Function and Morbidity

**DOI:** 10.3390/jcm15082884

**Published:** 2026-04-10

**Authors:** Rocío Segura Ruiz, José Miguel Reyes-Martínez, Emilia Priego-Cubero, Luna López-Coleto, Claudia Rivas-Cruces, Aurora García-Arcos, Francisco J. Labrador-Rodríguez, Nicola Lamberti, Fabio Manfredini, Pablo J. López-Soto

**Affiliations:** 1 Maimonides Institute of Biomedical Research of Córdoba (IMIBIC), Department of Nursing, Pharmacology and Physiotherapy, University of Córdoba, Reina Sofia University Hospital, 14004 Cordoba, Spain; rocio.segura.sspa@juntadeandalucia.es (R.S.R.); l32remaj@uco.es (J.M.R.-M.); luna.lopez@imibic.org (L.L.-C.); claudiarivascruces@gmail.com (C.R.-C.); n82gaara@uco.es (A.G.-A.); franciscoj.labrador.sspa@juntadeandalucia.es (F.J.L.-R.); 2 Maimonides Institute of Biomedical Research of Córdoba (IMIBIC), Reina Sofia University Hospital, 14004 Cordoba, Spain; 3 Department of Neuroscience and Rehabilitation, University of Ferrara, Via Luigi Borsari 46, 44121 Ferrara, Italy; lmbncl@unife.it (N.L.); fabio.manfredini@unife.it (F.M.)

**Keywords:** osteoporotic fractures, osteoporosis, exercise therapy, rehabilitation, physical functional performance, quality of life, accidental falls, mortality, systematic reviews as topic, meta-analysis as topic

## Abstract

**Background/Objectives:** Fragility fractures cause disability, independence loss, and death in older adults with osteoporosis. While exercise rehabilitation is recommended, its effectiveness and consistency in improving function, quality of life, and outcomes after a fracture remain uncertain. This study aimed to systematically assess the impact of structured exercise programs on physical function, quality of life, falls, morbidity, and mortality among adults recovering from fragility fractures. **Methods:** This review and meta-analysis followed PRISMA guidelines, with protocol registration in PROSPERO (CRD42024503933). MEDLINE, PubMed, EMBASE, Cochrane Library, CINAHL, and Web of Science were searched. Randomized controlled trials and quasi-experimental studies including adults (mean age ≥ 50 years) with fragility fractures were eligible if they evaluated structured exercise programs lasting ≥8 weeks compared with usual care or minimal intervention, and reported physical function, quality of life, falls, mortality, or morbidity outcomes. Risk of bias was assessed following the Cochrane Handbook for Systematic Reviews of Interventions guidelines. Primary outcomes comprised physical function, strength, balance, and health-related quality of life. Random-effects meta-analysis was applied where data were comparable. **Results:** Twenty-six studies with 4142 participants were included (*n* = 4142; ~80.4% women; mean age ~74.83 years). Interventions were mainly multicomponent (strength, balance, mobility, and functional training). Exercise improved physical function outcomes. At 12 months, pain measured by QUALEFFO-41 decreased (MD −11.61; 95% CI −22.99 to −0.23). Effects on strength, balance, and cognitive outcomes were inconsistent. **Conclusions:** Exercise-based rehabilitation after fragility fractures may improve physical function and reduce pain, but pooled effects are inconsistent and often highly heterogeneous. Evidence for effects on falls and mortality is sparse and does not support firm conclusions. Physical function measures may be the most practical primary endpoint for evaluating these interventions, interpreted cautiously. Funding: PIGE-0040-2020.

## 1. Introduction

In recent years, the aging population has increased in clinical conditions primarily affecting older adults. Osteoporosis is notable among the conditions with the highest morbidity and mortality rates. According to existing epidemiological data in Europe, in 2019, approximately 2,945,000 people suffered from osteoporosis, with 79.2% of them being women. Osteoporosis is recognized as a major public health problem, particularly in ageing populations [1]. Although osteoporosis does not produce significant symptoms, the fragility characteristic of the disease can result in bone fractures from minor or low-impact trauma. The primary sites are the wrist, hips, and vertebrae. Such fractures entail a range of adverse effects that affect individuals’ physical and emotional health, thereby impacting their quality of life. The pain and loss of mobility that ensue lead to a decrease in autonomy and independence, which increases the likelihood of anxiety, depression, or self-esteem issues. Similarly, these events are linked with a significant rise in morbidity and mortality. Approximately 1 in 5 patients may die within a year of a hip fracture [2].

Frailty—understood as a pathophysiological state that predisposes individuals to a greater vulnerability to disease and adverse effects—arises from an age-related lack of compensatory mechanisms and loss of homeostasis, resulting from an imbalance of body systems along with a decrease in functional reserve. Fragility fractures pose significant challenges to the health and mobility of those affected, particularly among older adults, and require coordinated post-fracture care [3,4,5]. Frailty and reduced physiological reserve are common after hip fractures and are associated with higher risk of adverse outcomes [6].

Consequently, exercise-based interventions have become crucial for treating and rehabilitating these fractures, offering multiple benefits for both physical recovery and overall well-being [7,8] and reducing side effects linked to other systems, such as the cardiac system [9,10,11]. For individuals recovering from fragility fractures, personalized exercise programs play a key role in regaining strength, enhancing balance and mobility. Weight-bearing exercises and resistance training contribute to bone density and muscle mass, addressing the underlying factors associated with fragility fractures and lowering the risk of subsequent incidents [12]. Conversely, balance and coordination exercises have proven particularly valuable in preventing falls, a common cause of fractures in frail individuals. Incorporating these exercises into rehabilitation programs aids individuals in regaining confidence in their movements, promoting independence and alleviating fear of falling [13]. Beyond physical benefits, exercise positively impacts mental health and quality of life. Regular physical activity can ease depression and anxiety often linked to the challenges of fracture recovery. Social and group exercise programs further help create a supportive environment, fostering community and motivation [14].

However, it is essential to tailor exercise prescriptions to individual abilities, considering comorbidities and overall health status. Supervised programs, guided by healthcare professionals or rehabilitation specialists, ensure that exercises are performed safely and effectively. Most published guidelines present similar recommendations, including prescribing personalized, multi-component exercise programs, discouraging prolonged sedentary behavior and inactivity, and combining exercise with optimal nutrition. To treat fragility, some authors recommend professionally supervised progressive resistance training [15].

Given these gaps and considering the impact of this issue on public health, it would be necessary to summarize the available evidence on the implementation of physical exercise interventions regarding health outcomes (well-being and quality of life) and aspects related to rehabilitation.

Previous evidence syntheses addressed exercise after specific fracture types. A prior systematic review focused on clinical trials in pelvic and/or lower-limb fragility fractures. It emphasized the present variability and reporting limitations across interventions [7]. A Cochrane review on interventions to improve mobility after hip fracture surgery studied postoperative hip fracture populations and mobility outcomes [12]. There have even been broader reviews that regarded exercise interventions in fragility fractures. However, a systematic review and meta-analysis that integrate randomized and quasi-experimental evidence across fragility fracture types, as well as follow-up time points, are still needed to clarify the effects of structured exercise on physical function, quality of life, and clinical outcomes.

The primary aim of this systematic review was thus to explore the existing scientific literature on the impact of physical exercise on individuals with fragility fractures. It assessed the effectiveness of exercise in reducing morbidity and mortality, its effects on strength, mobility, and balance, its potential to prevent future fractures, and its impact on quality of life.

## 2. Materials and Methods

A systematic review and meta-analysis were conducted in accordance with the criteria established by the PRISMA (Preferred Reporting Items for Systematic Reviews and Meta-Analyses) statement [16] (see Supplementary Table S1). A protocol was prospectively registered in PROSPERO (CRD42024503933). During the review, methodological refinements were introduced in response to the available evidence and formally documented through an updated PROSPERO amendment (9 February 2026). To ensure transparency, a “Protocol vs. Manuscript” reconciliation table (Supplementary Table S2) details the original specifications, final methodological decisions, rationale for changes, and their potential impact on the findings.

A comprehensive literature search was completed using the following electronic bibliographic databases: MEDLINE, PubMed, EMBASE, The Cochrane Library (Cochrane Database of Systematic Reviews, Cochrane Central Register of Controlled Trials (CENTRAL), Cochrane Methodology Register), CINAHL, and Web of Science (Science and Social Science Citation Index). All databases were searched until 16 January 2026. The search strategy only included terms relating to or describing the intervention and fragility fractures/osteoporosis (exercise; physical activity; fractures; bone; osteoporosis), connected through the Boolean operators “AND”/”OR” (see Supplementary Table S3 for full search strategy). Gray literature was also screened through research from institutional repositories or online platforms.

The criteria for study selection were established utilizing the Population, Intervention, Comparison, Outcome, and Study Design (PICOS) framework. (1) Population: Adults with a mean age of 50 years or older who had experienced a fragility fracture and received initial surgical or conservative treatment within the preceding year. Fragility fractures resulting from low-energy trauma, such as a fall from standing height or less, are a sign of underlying osteoporosis [17]. (2) Intervention: Any intervention consisting of a structured program of regular physical activity or exercise training lasting at least eight weeks, to ensure the intervention consisted of regular ongoing exercise training. (3) Comparison: The control group received standard care or did not participate in significant exercise training. Sham exercises, such as light stretching exercises, were allowed. Co-interventions with exercise training were permitted if the co-interventions were also administered to the control group. (4) Outcomes: primary endpoints included measures of physical functioning, strength, balance, cognitive functioning, or quality of life. Secondary outcomes included falls, mortality, and morbidity, defined as non-fatal adverse clinical events or complications reported during follow-up (e.g., postoperative complications, cardiovascular events, infections, or hospital admissions when specified). (5) Study Design: Randomized controlled trials (RCTs) or quasi-experimental studies published in English. This restriction to interventional designs focuses on studies that evaluate causal effects of structured exercise, minimizing confounding and selection bias inherent in observational designs for greater internal validity.

Studies were excluded if they met any of the following criteria: (1) lack of full-text availability; (2) interventions that only recommended or promoted physical activity, or targeted a single muscle group for reasons other than general fitness improvement; and (3) included participants with major medical conditions different from the fracture.

Multiple independent reviewers conducted the study selection process to ensure objectivity and consistency. Initially, two independent blinded reviewers carried out the screening and extraction process. Given the high number of records, four pairs of independent reviewers participated in the screening phase. Before starting, training was provided to standardize the inclusion criteria and ensure a uniform screening and extraction process. The review involved reading titles and abstracts to evaluate studies based on inclusion and exclusion criteria, followed by a full-text review for final inclusion. Articles not available in full text were excluded. Disagreements were resolved through discussion, with a third reviewer providing the final consensus.

The following data were extracted: identification of the authors, year of publication, study country, study design, characteristics of the participants in both the intervention and control groups (including age and sex; sample size; inclusion and exclusion criteria; sampling process), details of the intervention (type, timing, frequency, duration, and intensity), the control condition, and all measures related to outcomes; as well as information for the assessment of the risk of bias. Outcomes were extracted at all reported follow-up time points.

Two researchers independently assessed the quality of the included studies using the criteria described in the Cochrane Handbook for Systematic Reviews of Interventions. Risk of bias was assessed using the Revised Cochrane risk-of-bias tool for randomized trials (RoB2), in its 22nd August 2019 Version. RoB 2 evaluates five domains: (1) bias arising from the randomization process; (2) bias due to deviations from intended interventions; (3) bias due to missing outcome data; (4) bias in measurement of the outcome; and (5) bias in selection of the reported result. Each domain was judged as “Low risk of bias,” “Some concerns,” or “High risk of bias,” leading to an overall risk-of-bias judgment for each study. Any disagreements between the two researchers about the bias risk were resolved through discussion or consultation with a third researcher. Reporting bias was not assessed as fewer than 10 studies were available for all meta-analyses, which limited the effect of these methods. A list of all excluded full-text articles along with the reasons for exclusion was elaborated (see Supplementary Table S4).

During the elaboration of this manuscript, the authors used Elicit AI (Elicit, inc. (Update: 9 December 2025))to support Excel-based data extraction through data tabulation and cross-study outcome comparison—in order to facilitate outcome comparisons across the 26 included studies, screening, extraction, and the interpretation of information were performed by 2 human reviewers. The authors reviewed and edited all tool-assisted outputs and take full responsibility for the content of this publication. Figures, Tables, and Supplementary Materials were elaborated using Microsoft Word 2013, or Microsoft Excel 2013, with the exception of the PRISMA 2020 guidelines checklist supplement, which was downloaded from its official webpage and completed.

A narrative synthesis summarized the findings from the included studies, organized by their characteristics in relation to the measured outcomes. Specific outcomes (physical function, strength, balance, or quality of life) and characteristics of interventions were summarized. Studies were grouped by outcome domain and included when reporting comparable measures at the same follow-up timeline. A meta-analysis using a random-effects model was conducted using Review Manager v.5.4.1. Continuous outcomes were analyzed using standardized mean differences to estimate accuracy parameters across studies, though mean differences were used when measured on the same scale. All analyses were performed using the original reported data and scales. Data were extracted as reported, and no data transformation, imputation, recalculation, or standardization procedures were applied for the outcomes included in the meta-analyses. We evaluated heterogeneity using both the χ^2^ test and the I^2^ statistic, considering an I^2^ value over 50% as indicative of substantial heterogeneity. Sensitivity analyses were performed through the leave-one-out approach. No subgroup/meta-regression analyses were performed due to limited comparable studies per outcome.

Certainty of evidence for each outcome was assessed using the GRADE approach (Grading of Recommendations Assessment, Development and Evaluation) (see Supplementary Table S5).

## 3. Results

### 3.1. Study Selection and General Characteristics

**Figure 1:**
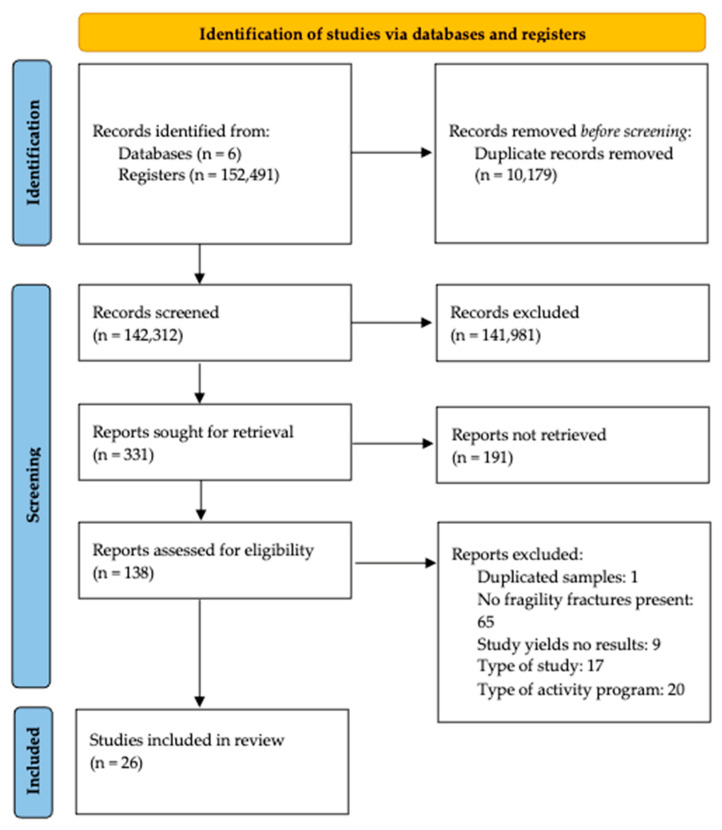
PRISMA flow diagram of study selection.

A total of 26 articles were selected for qualitative synthesis following PRISMA flow (Figure 1). Characteristics of the selected studies were extracted in Supplementary Table S6. The risk of bias of the selected articles is shown in Supplementary Table S7. A total of 4142 participants were involved across all included studies, with sample sizes ranging from 21 to 1314. Studies were conducted in 15 countries, predominantly in Europe, with some including Russia, North America, China, South Korea, Thailand, and Egypt. Twenty-five studies were randomized controlled studies (RCTs), and one was a non-randomized controlled trial [18].

A total of 13 studies focused on hip fractures [18,19,20,21,22,23,24,25,26,27,28,29,30], while 10 regarded vertebral fractures [31,32,33,34,35,36,37,38,39,40], and 3 included mixed fractures [41,42,43]—1 being wrist fractures of under 2 years, 1 including previous osteoporotic fracture, regardless of site, and 1 including a high-risk population for falls.

Regarding population, participants were predominantly older females. Sex distribution was similar in both groups. A total of 9 of the 26 studies were conducted on women-only samples, whereas one study had a bigger representation for men [18] and one other study included only male participants [21]. Mean female proportion was 80.4% across all studies, although it was registered as 74.4% mean female proportion for mixed-sex studies. Age eligibility thresholds were typically over 60 years or 65 years, with one study including patients from 42 years of age [33], with a mean age range of 66.5–81.5 years, and a mean age of around 74 years of age in both intervention and control groups. As a result, most participants were postmenopausal women suffering from osteoporosis presenting a previous history of fragility fractures. Eleven studies explicitly state exclusion criteria based on cognitive status, whether classified as “cognitive impaired” or evaluated through MMSE tests or Mini-cog test. No other reports on cognitive-impairment tests regarding the participant population were registered [20,21,22,23,24,25,26,28,33,36,38].

In relation to intervention type, 11 studies reported home-based delivery [18,20,22,25,27,28,31,36,39,41] with structured exercise sessions performed at home, and periodic professional home visits or follow-up calls, whereas 8 studies described supervised/therapist led delivery [19,28,29,31,34,37,38,40,42,43] ranging from fully supervised sessions to semi-supervised (supervised + home sessions), to unsupervised sessions with prior check-ins. Three studies reported a group-based format. Delivery categories were not mutually exclusive. Group-based interventions were described in 6 studies [32,34,37,38,40,41], comprising circuit-style exercise programs combined with educational programs. Additionally, 5 interventions were hospital-based early-phase rehab [18,19,21,23,27], then transitioned to home follow-up (including phone/telehealth-style follow-up).

The intervention program duration varied widely, ranging from 10 consecutive working days to 12 months, of which: (a) 8 studies lasted 12 months; (b) 1 study lasted 8 months; (c) 3 studies lasted 6 months; (d) 6 studies lasted 3 months; (e) 2 studies lasted 2 months; (f) 2 studies lasted 10 weeks; and (g) 1 study lasted 16 weeks.

Session frequency was described in 18 studies, and ranged from 1 to 6 sessions per week, though typically kept to 2–3 sessions/week, with a duration range of 30–120 min. In outpatient/community programs, supervised sessions commonly ranged from 1 session/week to 3 sessions/week. By contrast, early postoperative/inpatient interventions usually involved daily sessions.

### 3.2. Intervention Core Points

Interventions had multi-component approaches:

- Strength training in 16 studies focused on the lower limbs, using hip abductors, extensors, and leg press exercises to improve mobility and fall resilience, often with progressive resistance schemes or high-intensity programs.
- Balance and sensorimotor training in 13 studies included static and dynamic tasks, with some progressing from easy to more functional activities; one used behavioral change with follow-up.
- Mobility and gait training in 10 studies involved transfer practice, gait pattern training, and walking; one used a treadmill with body-weight support.
- Functional training was included in 8 studies, using sit-to-stand, steps, and daily movement tasks, integrated with strength and balance exercises.

### 3.3. Adherence Support

Telephonic follow-up and/or coaching were integrated into the intervention delivery or adherence support (e.g., scheduled calls, weekly motivational calls, therapist call-backs, and dietary consultations via telephone call) [18,20,21,22,36,39]. One study, however, integrated online chat via a WeChat app-based follow-up as an alternative to telephone contact after discharge in a balance-training program [21].

### 3.4. Outcomes

#### 3.4.1. Physical Function

Physical function was evaluated through mobility and/or performance tests, gait speed and resistance, ambulation scales, ADL, and function/disability questionnaires. TUG, 20 m walking speed, chair-stand, 6MWT, “community ambulation”, Koval, FAC, K-MBI, FIM, WOMAC, and Modified Oswestry were used to measure physical function [20,21,22,23,38,39,41,42].

**Figure 2:**
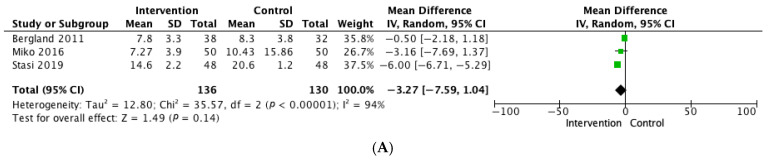

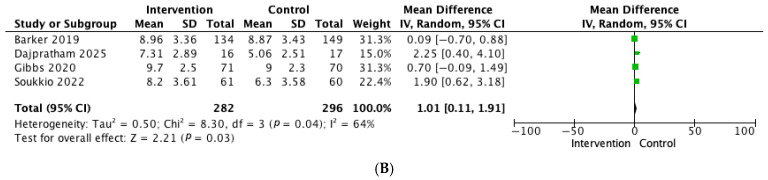
(**A**) Effect of exercise interventions on TUG (seconds) at 3 months: random-effects meta-analysis [27,34,42]. (**B**) Effect of exercise interventions on SPPB performance at 12 months: random-effects meta-analysis [24,30,33,36].

Timed Up and Go (TUG) tests showed significant improvement at 4 weeks, 3 months, and 12 months in 3 of the 6 studies that included this technique and reported ambulation as an outcome [20,27,34,39,42]. However, given the variability in timing and measurement units, the quantitative analysis of three studies [27,34,42] reporting TUG in seconds at three months showed that, although there was a reduction in seconds for the intervention group, it was not statistically significant (mean difference [MD], −3.27; 95% CI: −7.59–1.04; *p* = 0.14; I^2^ = 94%) (Figure 2A). Given the extreme heterogeneity, this pooled estimate should be interpreted with caution and considered exploratory rather than confirmatory. Sensitivity analyses employing a leave-one-out approach revealed substantial instability of the pooled effect, with heterogeneity markedly decreasing when specific studies were excluded and, conversely, statistical significance emerging when others were removed. These findings indicate that the overall effect is highly influenced by individual studies and does not reflect a consistent intervention effect across trials. No TUG reporting study met the overall criteria for high risk of bias, although performance bias was high due to the limitation of blinding participants and personnel in such interventions. Excluding the study by Stasi et al. [27] notably reduced heterogeneity (I^2^ = 14%) and lowered the pooled mean difference, whereas excluding Bergland et al. [34] yielded a statistically significant pooled effect with moderate heterogeneity. These findings suggest that overall outcomes are heavily influenced by the inclusion of specific studies. No TUG reporting study met the overall criteria for high risk of bias, although performance bias was high due to the limitation of blinding participants and personnel in such interventions.

Two studies found statistically significant differences at week 8 and at the 1-year follow-up, respectively. One study showed a −4.2 change in the TUG test after 8 weeks (95% CI −8.0 to −0.3), with a between-group difference of *p* = 0.05 at the 1-year control [20,42]. However, Papaioannou et al. and Gibbs et al. did not observe differences between groups at 6 and/or 12 months [39].

A 20 m Maximum Walking Speed was used by one study in a vertebral fracture cohort, showing significant improvement at both 3 and 12 months (baseline: Intervention −1.3 s vs. Control +0.6 s; *p* = 0.001 to 3 months; Intervention −0.9 s vs. Control +0.6 s; *p* = 0.019 to 12 months) [38]. Ambulation was measured using two methods: community ambulation for distances longer than 300 m and the 6 min walk test [22,41]. None of them registered significant differences. Another study [23] described the use of the Koval walking ability score and Functional Ambulation Category (FAC), recording significantly higher changes in its intervention group at 3 weeks, 3 months, and 6 months [23]. The hip-joint function score also showed significant differences at 2, 6, and 12 weeks, although no significance was observed during the first bedside standing [21]. And two studies assessed global physical performance using the Short Physical Performance Battery (SPPB), yielding heterogeneous results: one reported significant improvement following a long-term home-based exercise program [25], while the other found no consistent effects on performance [36].

Physical function was also evaluated using chair-rise or sit-to-stand (STS) tests, either independently or as part of the SPPB [24,33,36]. As these measures are not directly comparable across studies, only outcomes assessed with the same instrument at the same time points were pooled; all remaining tools (chair-rise, STS) were summarized narratively.

At 12 months, the intervention group showed a higher SPPB score compared with usual care (MD: 1.01 points, 95% CI 0.11 to 1.91; *p* = 0.04; I^2^ = 64%) (Figure 2B). Leave-one-out sensitivity analyses revealed moderate instability of the pooled estimate. Removal of Barker et al. [33] increased the effect size and reduced heterogeneity (MD 1.40; I^2^ = 49%), whereas the exclusion of Dajpratham et al. [30]—classified as high risk of bias—reduced the pooled estimate to non-significance (MD 0.78; *p* = 0.09; I^2^ = 64%). Similarly, removing Gibbs et al. [36] or Soukkio et al. [24] rendered the effect non-significant, with heterogeneity remaining moderate to high (I^2^ 57–76%). Overall, statistical significance was lost in three of four scenarios, indicating that the pooled effect is sensitive to both individual-trial variability and between-study variability. Given the presence of one high-risk-of-bias study [30] and frequent performance bias across trials, these findings should be interpreted cautiously and do not yet demonstrate a fully consistent benefit.

#### 3.4.2. Strength

Although strength training was included in many interventions, only five studies quantified strength outcomes [19,27,37,40,41]. Across all studies, strength was assessed using heterogeneous instruments, which made quantitative pooling unfeasible and required narrative synthesis.

The study written by Berg et al. described 1RM strength program with leg press and hip abduction following Maximal Strength Training (MST) measuring 1RM bilateral leg press, 1RM unilateral leg press (both fractured and healthy), and 1RM hip abduction. The bilateral leg press did not show any significant differences between intervention and control groups, though unilateral and hip abduction 1RM registered significant differences where the intervention group showed a stronger fracture side of the body compared to control group [19]. Stasi et al. also reported significant increases in the isometric strength of fractured-side abductors in the intervention group [27].

As described by the OsteoACTIVE exercise program [41], Isokinetic Quadriceps Strength measured left and right peak torque and total work. These, however, did not show significant changes between groups, even though there was a slight upwards trend in the intervention group [41]. Another study focusing on physiotherapy training involving trunk and lower-limb strength found improvement in back extensors in their intervention group (from week 0 to week 10), as well as changes in abdominal flexors and quadriceps [37].

One study included hand force as a measurable variable and included chair stand repetitions as well, but found no clear superiority of one group over the other [40]. Two more studies included strength-based exercises in their interventions but did not report them as quantified outcomes [21,25].

Several trials reported multiple strength comparators (like unilateral vs. bilateral assessments). Considering the risk of multiplicity, overall patterns were emphasized rather than establishing single comparisons, as isolated statistically significant results have to be interpreted cautiously. Overall certainty was limited by heterogeneity and performance bias.

#### 3.4.3. Balance

Balance outcomes were assessed with heterogeneous instruments across all studies, making quantitative pooling impossible, thus requiring narrative synthesis.

Two studies measured balance using the Romberg one-leg stand with eyes open and closed, on both legs, as well as tandem walking. One-leg stand showed a statistically significant worsening with open eyes, whereas it showed a statistically significant improvement with closed eyes (*p* = 0.040 for the right leg and *p* = 0.026 for the left leg). This renders these findings inconsistent, and perhaps reflects variability in measurements rather than an effect. It should, therefore, be interpreted cautiously. Tandem walking showed a statistically significant difference when walking backwards (*p* = 0.027). Although not significant for forwards tandem walking (*p* = 0.068) [40,41].

Berg et al. describe the use of the Unipedal Stance Test to assess balance. The intervention group shows a non-significant statistical difference compared to the control group (*p* = 0.54 post-intervention and *p* = 0.22 after 6 months) [19].

The Four-Square Step Test (FSST) was referenced in one study where intervention group showed a non-significant difference between intervention groups (*p* = 0.767) [41], while studies measuring through the Functional Reach Test (FRT) reported a statistically significant difference for intervention and control groups on week 8 (95% CI 1.0 to 7.8 for FRT on one study and a difference of 3.8 (1.5), *p* = 0.001 on another) [20,38]. Olsen and Bergland, however, registered a statistically significant difference after 3 months, which decreased to a non-significant difference at the 12-month control (Intervention 2.8 SD (4.1) vs. Control 2.2 SD (2.3) → diff 0.7 (1.2), *p* = 0.49) [38].

The Berg Balance Scale was used in two studies that showed a statistically significant difference between control and intervention groups at the 12-week and 1-year controls (Intervention: first bedside standing 17.46 ± 1.28 > 2 w 33.59 ± 0.80 > 6 w 46.95 ± 1.72 > 12 w 49.29 ± 2.40 vs. Control: 17.67 ± 1.26 > 25.38 ± 1.68 > 35.60 ± 1.31 > 41.55 ± 2.31 showing a *p* < 0.05) [21,42].

Finally, a balance test on a platform was carried out in one study, with a baseline for the intervention group of 20.6 (17.9; 23.5) vs. control group of 22.0 (20.7; 25.0), with *p* = 0.22. Thus, no statistically significant difference was found [37].

Balance findings were heterogeneous in measurement and outcome comparisons across trials. Thus, isolated statistically significant results should be interpreted with caution.

#### 3.4.4. Quality of Life

Twelve studies evaluate quality of life as an outcome [20,23,26,28,33,34,35,36,37,39,40,41] using a wide range of instruments, which limits direct comparability across trials. Four of these studies used the QUALEFFO-41 scale [33,34,35,40] to measure quality of life, while three studies used the EQ-5D scale, two studies used the SF-36 scale, and other studies used scales like the GHQ-20, Osteoporosis Quality of Life Questionnaire (OQLQ) and/or Mini-OQLQ, sickness impact profile (SIP), 15D, Assessment of Quality of Life (AQoL), or the self-assembled Quality of life questionnaire [37].

Among vertebral fracture populations, Bergland et al. reported meaningful enhancements in QUALEFFO-41 domains of pain (*p* = 0.005), physical function (*p* = 0.047), and mental function (*p* = 0.04) at 12 months, accompanied by short-term psychological gains on the GHQ-20 [33]. Similarly, Evstigneeva et al. observed improvements between groups across QUALEFFO-41 domains—including pain, mobility, social function, and general health perception—after a 12-month supervised exercise program [35], whereas Barker et al. found no significant between-group differences for QUALEFFO-41 or EQ-5D-5L scores in their study [33]. In contrast, Papaioannou et al. registered consistent improvements in OQLQ domains of symptoms, emotions, leisure, and daily activities at both six and twelve months [39], while Gibbs et al. reported no statistically significant between-group differences in mini-OQLQ, EQ-5D, or HUI indexes [36]. Regarding quality-of-life instruments, Hakestad et al. detected no significant change in physical or mental scores [38], whereas Lee et al. [20] showed significant improvements in the Physical Component Summary (*p* = 0.017) from their multicomponent home-based rehabilitation. Though no differences in mental health were recorded. Similarly, Oh et al. [23] observed short-term improvements in EQ-5D favoring the antigravity treadmill group at three weeks (*p* = 0.005), but these were lost at later follow-ups. Spångeus et al. [40] found improvements in RAND-36 and QUALEFFO-41 social function domains (*p* < 0.05) that diminished by the end of year one, and Soukkio et al. [26] reported no between-group differences in Health-Related Quality of Life (HRQoL) over 12 months. Taylor et al. [28] observed no significant changes between groups in AQoL-8D scores, while Malmros et al. [37] noted progressive, statistically significant QoL gains in the physiotherapy group from week 5 (*p* = 0.01) through week 22 (*p* = 0.0008). All in all, these findings were mixed. While some studies reported improvements in HRQoL domains (specifically, pain, and mobility), not all studies observed differences between groups, and even when differences were observed, they were not consistent across follow-up time points. Therefore, conclusions should be taken with caution.

**Figure 3:**
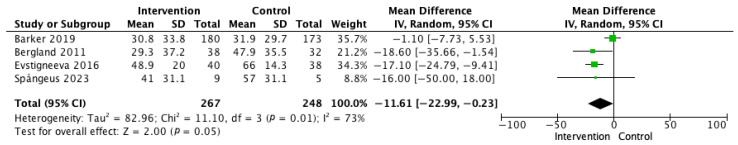
Effect of exercise interventions on pain (QUALEFFO-41) at 12 months [33,34,35,40].

Regarding quantitative analysis, at 12 months, four exercise interventions [33,34,35,40] indicates possible reduced pain levels (QUALEFFO-41) compared to the control (MD −11.61, 95% CI −22.99 to −0.23; *p* = 0.05; I^2^ = 73%), showing improvement despite heterogeneity. (Figure 3). However, sensitivity analyses using a leave-one-out approach revealed significant instability of the pooled effect estimate. Removing Barker et al. [33] resulted in a larger pooled mean difference and complete elimination of heterogeneity (I^2^ = 0%), indicating this study was the main source of between-study variability. Conversely, excluding Evstigneeva et al. [35] rendered the pooled effect non-significant, suggesting that this study largely influenced the overall effect. Although most studies contributing to this analysis were judged as low risk or some concerns, one study included in the broader review was classified as high risk of bias; however, it did not materially drive the pooled QoL estimate. As such, evidence for sustained pain-related HRQoL is not consistent.

#### 3.4.5. Independence/ADL

A study using the Korean Modified Barthel Index (K-MBI) found significant changes after 6 months (increase of 10.7, 95% CI 4.6–16.8; *p* = 0.001) since the beginning of the intervention—with no statistical significance at 3 weeks and 3 months, while the Functional Independence Measure (FIM) registered a significant change from baseline to 12 months since the start of the intervention (95% CI 0.5–8.5; *p* = 0.029) [23].

On a more auto-perceived level, WOMAC (Western Ontario and McMaster Universities Osteoarthritis Index) score results indicated better auto-perceived results regarding function in all time windows—<3 months (67 ± 23 vs. 84 ± 17 (*p* < 0.001)); 3–6 months (42.8 ± 15.9 vs. 50.2 ± 14.9 (*p* = 0.02)); 6 months-1 year (24.3 ± 12.9 vs. 40.0 ± 13.1 (*p* < 0.001)) [18] in the intervention group. Another study utilized the Modified Oswestry Questionnaire, which reported no significant differences in total score between groups, but showed improved function and no worsening in its intervention group [37].

#### 3.4.6. Falls

One study assessed falls as events, reporting fall counts and analyzing the time until each event occurred. The multifactorial clinic intervention reduced total falls (IRR 0.72; 95% CI 0.63–0.83; *p* < 0.001) and injurious falls (IRR 0.59; 95% CI 0.47–0.74; *p* < 0.001), while also reducing fractures (IRR 0.49; 95% CI 0.29–0.83; *p* = 0.008) and fall-induced injuries (IRR 0.45; 95% CI 0.32–0.64; *p* < 0.001). Benefits were also observed for time to first fall (HR 0.82; 95% CI 0.71–0.96; *p* = 0.012) and time to first injurious fall (HR 0.65; 95% CI 0.52–0.81; *p* < 0.001) [43]. This evidence, however, is extracted from a single trial. It should not be interpreted as definite proof of fall reduction, as certainty is low.

A second study reported a “relative risk of falling” following a balance training program, indicating a lower risk in the intervention group (0.12) compared with the control (0.229), with statistical significance (*p* < 0.05) [42].

Two studies assessed falls using falls-related fear-of-falling scales. In a progressive balance training program, the Falls Efficacy Scale increased significantly in the intervention group compared with the control group at 2, 6, and 12 weeks (*p* < 0.001 at all reported time points), although no baseline differences were observed [21]. In contrast, another study using the FES-I reported no changes, with values similar at baseline, post-intervention, and at 1-year follow-up [40].

Falls outcomes were highly heterogeneous, as they were measured both as incident events (IRR/HR) and as self-efficacy perceptions (FES/FES-I), making direct comparability across studies not feasible. Fall reduction effects are still inconclusive.

#### 3.4.7. Morbidity/Mortality

Eight studies evaluated the impact of their interventions on morbidity—operated as non-fatal adverse clinical events/complications reported by the included studies, which were heterogeneously defined—mortality and subsequent hospital admissions during or after their interventions [18,20,22,24,28,33,41,43], though only one [18] study reported mortality as a primary outcome. The study registered a one-year mortality rate of 43.3% in its control group. Meanwhile, the intervention group had a one-year mortality rate of 26.6%, which was significantly lower than that of the control group (*p* = 0.04). Mortality rate in the 30-day period was registered as 21.7% for the control group, and 9.4% for the intervention group (*p* = 0.01), which also saw a significant reduction in mortality. Most of these deaths (33.3% for control group, 12.5% for intervention group) occurred in the first 3 months since the beginning of the program, with a decrease in mortality directly related to time passed (8.3% before the 6-month mark vs. 1.7% before the year mark) for the control group, while the intervention group observed 1.6% mortality before the 6-month mark and 12.5% before the year mark. However, 30-day period deaths are included in the 3-month mark category [18]. Survival rate at the 1-year mark proved to be significantly lower in the control group.

Subsequent hospital admissions were not consistently reported across studies, though one study evaluating a post-hip fracture walking program found no significant differences between groups in hospital admissions during its intervention period [28]. The remaining studies did not provide data on hospital admissions.

Regarding morbidity, postoperative problems were reported in one study [18], which described that they were present in 36.7% of the control group participants, compared to 18.8% of participants from the intervention group (*p* = 0.03). Other specific events were recorded [18], and while heart attacks were significantly more present among the control group participants (20.0% for the control group vs. 4.7% for the intervention group; *p* = 0.01), chest infections, UTIs, DVT, depression, or strokes did not show any significance regarding differences between these groups [18]. Other adverse events were reported in several studies, mostly related to safety. A trial on patients with osteoporotic vertebral fractures reported falls, fragility fractures, cardiovascular events, and infections in its intervention groups, though no serious adverse events were linked to the interventions, and no differences between groups were registered [30]. In the same fashion, a walking intervention trial on the hip-fracture population reported no serious adverse events related to the intervention, and no between-group differences [28]. As secondary outcomes, morbidity (non-fatal adverse clinical events/complications) and mortality were reported heterogeneously across studies.

#### 3.4.8. Cognitive Functioning

Eight studies evaluated cognitive function using either direct neuropsychological tests or quality-of-life scales. Regarding global cognition, Oh et al. found no significant differences using the K-MMSE (*p* = 0.211) [23], whereas Soukkio et al. reported a significant 4.5-point improvement in the Functional Independence Measure (FIM) cognitive scale at 12 months (*p* = 0.029) [25]. However, a post hoc analysis of the same cohort showed no significant benefit on the 15D mental function dimension (*p* = 0.50) [26]. In patients with vertebral fractures, a study observed sustained improvements in the QUALEFFO-41 mental function domain (*p* = 0.04) and short-term reductions in psychological distress (*p* = 0.009), though these effects disappeared after 1 year (*p* = 0.17) [34]. In contrast, several trials reported no significant differences between groups regarding mental health, including Barker et al. (*p* = 0.19) [33], Evstigneeva et al. (*p* = 0.093) [35], and Spångeus et al. (*p* = 0.07) [40]. Similarly, Lee et al. found no significant impact on depressive symptoms at baseline values (*p* = 0.201) [20]. Overall, evidence supporting cognitive benefits remains insufficient and inconsistent.

Reporting bias could not be assessed for most outcomes, since fewer than 10 studies were available per meta-analysis. Likewise, no subgroup/meta-regression analyses were performed as there were limited comparable studies per outcome.

According to GRADE, the certainty of evidence was moderate for TUG (<3 months), SPPB (12 months), and pain (QUALEFFO-41, 12 months) (See Supplementary Table S4).

According to the RoB 2 assessment, 16 studies were judged as low risk of bias, 8 as having some concerns, and 2 as high risk of bias. High-risk judgments were mainly driven by non-randomized design and substantial attrition. Performance bias due to lack of participant blinding was frequent but considered inherent to exercise interventions (See Supplementary Table S7).

## 4. Discussion

Overall, the findings of the present systematic review suggest that structured exercise interventions following a fragility fracture may be associated with improvements in physical function and mobility. Nonetheless, the observed effects were heterogeneous and, in some outcomes, unstable. As such, conclusions regarding clinical significance should be interpreted with caution. This supports the vital role of post-fracture rehabilitation that previous literature proposed [8,12].

Across fracture types, functional outcomes—including gait, mobility, physical performance, and balance—reported improvements in certain measures. The improvements in Timed Up and Go, walking speed and ambulation measures were reported in multiple studies [20,22,23,38], although the findings were heterogeneous across interventions and time points, with balance being non-significant.

Although strength training was incorporated into most intervention programs, strength outcomes were highly variable, with some studies reporting improvement in fracture-side, unilateral strength [19,27], whereas other studies did not show differences between groups [40,41]. Current evidence thus does not agree on the role of strength training in functional recovery.

Regarding balance, some studies reported improvements when assessed with functional scales—such as the Berg Balance Scale or Timed Up and Go [2,21,23]—whereas static or device measures yielded less significant findings [19,38]. Overall, the balance findings were mixed.

The effects that exercise had on quality of life varied depending on the instrument and domain assessed. Pain and physical function domains showed improvements in several studies [34,35,37,39], whereas others found no significant differences between groups in overall health-related quality of life [26,33,36], which is consistent with previous literature highlighting variability across HRQoL measures [8].

Evidence on cognition was limited, as only a small number of studies assessed it, yet they were inconsistent. While some trials reported short-term improvements, others found no sustained or even significant differences. Thus, current evidence does not support a consistent effect on cognition or mental health.

Regarding falls, studies that evaluated falls as clinical events reported a pattern of reductions in fall incidence and/or fall risk after intervention [42,43]. Nevertheless, these findings are based on a reduced number of trials and do not sustain a significant conclusion. In addition, fear of falling showed inconsistent results across studies [21,40].

Concerning mortality and morbidity, the evidence was limited, as only a few of the studies included assessed these outcomes. There was a significant reduction in early and one-year mortality following an exercise-based intervention during the early period after fracture, which indicates there might be a critical time window that impacts clinical outcomes far beyond functional recovery, even though very few studies were included [18]. However, the inconsistent data on morbidity and hospital admissions, as well as the inconsistency in its definition, strongly suggest that the current evidence is insufficient and that future trials are needed to address this.

Despite the heterogenous methodology and broad variations in both interventions and outcomes, the overall findings suggest that there might be benefits to the implementation of individualized exercise programs that follow the guidelines described in previous literature [8,12]. Nonetheless, the findings are mixed and certainty is uneven across outcomes.

Physical function was one of the most common outcomes to exercise-based interventions following fragility fractures, as it showed earlier, more frequent improvements than other domains. These were repeatedly observed in measures such as the Timed Up and Go test, walking speed, and ambulation scales across the vast majority of trials assessing these outcomes [20,22,23,38]. Multicomponent functional tests, particularly the Timed Up and Go and the Short Physical Performance Battery, may better capture changes than isolated measures, likely because they integrate balance, strength, and mobility demands. However, the results for the SPPB were heterogeneous, with some studies reporting improvements after longer or progressive interventions [24], while others found no consistent effects [36], which limits confidence in a sustained effect. Overall, these findings suggest that improvements in physical function should be interpreted with caution, as their consistency and clinical significance are not demonstrated, even though for some measures (e.g., SPPB) a pooled mean variation approaching the minimal clinically important difference was observed, which is interesting but still a preliminary result.

There is marked methodological heterogeneity across the included studies, with a wide variability in intervention duration, which ranges from days to a year, session frequency and intensity, and the wide difference in outcomes and assessment instruments. This variability limits direct comparability between trials and limits the feasibility of conducting a robust meta-analysis across several domains. Differences in intervention intensity and progression, combined with the wide variability of measures, contribute to a broader range of observed effects. Consistently, sensitivity analyses across all meta-analyses showed that pooled effect estimates and heterogeneity were highly dependent on the inclusion of individual studies, with substantial changes in both magnitude and statistical significance. Consequently, the results should be interpreted as exploratory, rather than definitive.

Several limitations were identified in the present systematic review. First, although most trials were judged as having a low risk of bias, two studies were classified as high risk of bias and eight as having some concerns, mainly due to issues related to randomization reporting or missing outcome data. Second, studies on outcomes like mortality, falls, or cognition were scarce, limiting conclusions. Excluding observational studies might have omitted real-world evidence on long-term outcomes like falls, hospitalizations, and mortality. Third, the marked heterogeneity in outcome measures and assessment instruments hinders direct comparability across studies and complexifies a synthesis of results. In line with this, the instability observed in sensitivity analyses emphasizes the influence of clinical and methodological variability on pooled estimates. Additionally, although the search was conducted without language restrictions, the final inclusion of English-language studies only—adopted for feasibility and data extractability reasons—may have introduced language bias. Fourth, most study populations were predominantly female, which, on the one hand, demonstrates the epidemiological tendency towards fragility fractures, but also limits the generalizability of these findings to males. Moreover, the different inclusion criteria across studies may have indirectly affected the morbidity and mortality outcomes in the comparisons. It should be noted that morbidity was heterogeneously defined across studies, and primarily referred to non-fatal clinical complications rather than standardized composite endpoints. Fifth, the lack of standardization in exercise dose, intensity, duration, frequency, and progression restricts the capacity to identify optimal intervention parameters and might even contribute to the variability of the results gathered [8,12]. In addition, minor discrepancies between the initial PROSPERO registration and the final manuscript were identified during the peer review. These mainly concerned the clarification of eligible study designs, outcome prioritization, specification of risk-of-bias tools, and reporting enhancements (e.g., GRADE application). All differences are transparently documented in Supplementary Table S2, and the PROSPERO record has been updated accordingly. These refinements reflect methodological clarification and feasibility considerations rather than post hoc outcome-driven modifications, and they do not alter this review’s conceptual scope or overall conclusions.

## 5. Conclusions

The findings of the present review suggest that exercise might play an important role in rehabilitation following fragility fractures. Although the gathered evidence is heterogeneous, functional outcomes, especially those related to mobility, were among the most frequently regarded domains and often reported improvements following intervention—which supports using them as priority endpoints.

Sensitivity analyses across meta-analyses showed that pooled effects varied in size and significance and were sometimes influenced by individual studies, highlighting the need for cautious interpretation, and, more importantly, the exploratory nature of these findings. Moreover, isolated significant findings should not be interpreted as consistent evidence of benefit.

Future research should prioritize RCTs that incorporate relevant clinical outcomes, such as mortality and hospitalizations, as well as standardized measures of physical function, in order to improve comparability between studies. Follow-up periods should be kept to a 1-to-2-year period to determine whether these functional benefits are maintained over time. Finally, a greater inclusion of patients diagnosed with cognitive impairment, as well as an adequate male representation, in order to better support evidence for this group, may help to better reflect the impact of these programs on cognition. Addressing these issues would strengthen the evidence base and offer more precise and stable programs after fragility fractures.

## Data Availability

All data analyzed in this study are sourced from previously published articles, which are cited in the reference list. Structured extraction tables and risk-of-bias assessments are available in the Supplementary Materials. Further methodological details are available from the corresponding authors upon reasonable request.

## Supplementary Materials

The following supporting information can be downloaded at: https://www.mdpi.com/article/10.3390/jcm15082884/s1. Table S1: PRISMA 2020 checklist; Table S2: Protocol vs Manuscript Reconciliation; Table S3: Search strategy per database; Table S4: List of full-read articles excluded with reason for exclusion and DOI code/article title for identification; Table S5: GRADE rating; Table S6: Full data extraction sheet; Table S7: Risk of bias assessment per study and domain.

## Abbreviations

The following abbreviations are used in this manuscript:

15D	Fifteen-dimensional health-related quality of life instrument
1RM	One-repetition maximum
6MWT	Six-minute walk test
ADL	Activities of daily living
AQoL	Assessment of Quality of Life
AQoL-8D	Assessment of Quality of Life-8 Dimension
CC BY	Creative Commons Attribution
CENTRAL	Cochrane Central Register of Controlled Trial
CI	Confidence interval
CINAHL	Cumulative Index to Nursing and Allied Health Literature
CRediT	Contributor Roles Taxonomy
DVT	Deep vein thrombosis
EMBASE	Excerpta Medica database
EQ-5D	EuroQol 5-Dimension questionnaire
EQ-5D-5L	EuroQol 5-Dimension questionnaire-5-Level version
FAC	Functional Ambulation Category
FES	Falls Efficacy Scale
FES-I	Falls Efficacy Scale International
FIM	Functional Independence Measure
FRT	Functional Reach Test
FSST	Four-Square Step Test
GHQ-20	General Health Questionnaire-20 items
HR	Hazard ratio
HRQoL	Health-related quality of life
HUI	Health Utilities Index
I^2^	I-squared heterogeneity statistic
IRR	Incidence rate ratio
K-MBI	Korean Modified Barthel Index
K-MMSE	Korean Mini-Mental State Examination
MD	Mean difference
MEDLINE	Medical Literature Analysis and Retrieval System Online
Mini-Cog	Mini-Cog cognitive screening test
Mini-OQLQ	Mini version of the Osteoporosis Quality of Life Questionnaire
MMSE	Mini-Mental State Examination
MST	Maximal Strength Training
OQLQ	Osteoporosis Quality of Life Questionnaire
PICOS	Population, Intervention, Comparison, Outcomes, and Study design
PRISMA	Preferred Reporting Items for Systematic Reviews and Meta-Analyses
PROSPERO	International Prospective Register of Systematic Reviews
PubMed	PubMed database
QoL	Quality of life
QUALEFFO-41	Quality of Life Questionnaire of the European Foundation for Osteoporosis-41 items
RAND-36	RAND 36-Item Health Survey
RCT	Randomized controlled trial
SD	Standard deviation
SF-36	36-Item Short Form Health Survey
SIP	Sickness Impact Profile
SPPB	Short Physical Performance Battery
STS	Sit-to-stand
TUG	Timed Up and Go
UTI	Urinary tract infection
WHO	World Health Organization
WOMAC	Western Ontario and McMaster Universities Osteoarthritis Index
