# Supplementary material for: Physical Exercise After Fragility Fractures: A Systematic Review and Meta-Analysis of Function and Morbidity
Source: J Clin Med. 2026 Apr 10;15(8):2884. doi: 10.3390/jcm15082884 (PMC13116814; doi:10.3390/jcm15082884)
Supplement: Supplementary file 1 [file jcm-15-02884-s001.zip › Table S2. Protocol vs Manuscript Reconciliation.pdf]

Item	PROSPERO (CRD42024503933)	Final manuscript	Reconciliation / what changed	Rationale / transparency note
Review team / authorship (named investigators vs manuscript authors)	Listed review team members: Pablo Jesús López-Soto; Juan de la Cruz López-Carrasco; Rocío Segura Ruiz; Claudia Rivas-Cruces; Nicola Lamberti; Fabio Manfredini; Giovanni Piva	Manuscript author list: Rocío Segura Ruiz; José Miguel Reyes-Martínez; Emilia Priego-Cubero; Luna López-Coleto; Claudia Rivas-Cruces; Aurora García-Arcos; Francisco J. Labrador-Rodríguez; Nicola Lamberti; Fabio Manfredini; Pablo J. López-Soto	Added to manuscript (not listed in PROSPERO): José Miguel Reyes-Martínez; Emilia Priego-Cubero; Luna López-Coleto; Aurora García-Arcos; Francisco J. Labrador-Rodríguez. Not included as manuscript authors (listed in PROSPERO): Juan de la Cruz López-Carrasco; Giovanni Piva.	The final author list reflects contributors who actively participated in study conduct and manuscript production and meet journal authorship criteria. According to ICMJE criteria, Individuals listed in PROSPERO but not in the final manuscript did not contribute sufficiently to qualify for authorship in the completed review and/or were not involved in the final manuscript, and are, therefore, not included as authors in the submitted manuscript.
Language restriction	"No language restrictions will be applied at the search stage[…]" ; "however… only studies published in English will be included in the final synthesis."	Eligibility explicitly restricts included studies to those published in English ("…RCTs or quasi-experimental studies published in English").	There is no change in the restriction (English-only inclusion). The only discrepancy is inaccuracy: PROSPERO states no search-stage restriction + English-only inclusion, while the manuscript only states the English-only inclusion criterion.	PROSPERO imposed no search-stage restriction but limited synthesis to English only. The manuscript clarifies this as a specified English-language eligibility criterion, due to feasibility and data extraction reliability, not a post hoc restriction. It was applied before final inclusion, and language bias is acknowledged as a limitation.

Item	PROSPERO (CRD42024503933)	Final manuscript	Reconciliation / what changed	Rationale / transparency note
Eligible study designs	Randomized controlled trials (RCTs) and quasi-RCTs (randomized / quasi-randomized allocation).	RCTs or quasi-experimental studies, and non-randomized controlled designs (controlled clinical trials / non-randomized controlled trials).	Broadened eligible designs from RCT/quasi-RCT (protocol) to include non-randomized controlled (quasi-experimental) designs in the manuscript.	This change was made to avoid excluding relevant controlled evidence in an area with limited RCTs.
Outocome priorization and definitions	Emphasis on morbidity/clinical outcomes	Greater prioritization of physical function and quality of life, with explicit operational definition of "morbidity."	Reprioritization occurred after recognizing inconsistent reporting of mortality and long-term clinical endpoints. Functional recovery and patient-reported outcomes are the most consistently measured and clinically relevant outcomes in post-fragility fracture rehabilitation.	The protocol emphasized morbidity and clinical endpoints. During extraction, these outcomes were inconsistently reported across studies. Functional and quality-of-life measures were more systematically available and clinically relevant. Outcomes were reprioritized and "morbidity" operationally clarified. This enhances interpretability without altering objectives.
Search strategy description	Core concepts focused on exercise interventions and fracture populations.	Expanded and refined strategy incorporating additional population-specific descriptors (e.g., fragility fracture, osteoporotic fracture, osteoporosis).	Methodological refinement to optimize comprehensiveness and precision. Improve sensitivity while reducing irrelevant retrieval, ensuring that all clinically relevant controlled studies in fragility fracture rehabilitation were captured without altering the conceptual framework.	Population-related terms like fragility fracture and osteoporosis were added to improve search sensitivity and precision. The conceptual domains stayed the same. The refinement was methodological, not outcome-driven, enhancing reproducibility and accuracy. It did not affect scope or conclusions.

Item	PROSPERO (CRD42024503933)	Final manuscript	Reconciliation / what changed	Rationale / transparency note
Risk-of-bias Plan and reporting	Risk-of-bias assessment was planned, although the specific instrument was not explicitly detailed.	The Cochrane Risk of Bias 2 (RoB 2) tool was applied for randomized trials, with structured domain-level judgments and overall risk classification	Explicit specification of the assessment instrument and reporting framework used in practice. To align the review with current Cochrane methodological standards and enhance transparency, reproducibility, and interpretability of internal validity assessments.	The clarification strengthens methodological rigor without modifying eligibility criteria, data synthesis, or conclusions.
Certainty assessment (GRADE)	Certainty of evidence assessment was not explicitly specified in the registry entry.	The GRADE framework was systematically applied to all key outcomes to rate certainty (risk of bias, inconsistency, indirectness, imprecision, publication bias).	Post-protocol methodological enhancement incorporated before final interpretation. To align with contemporary evidence synthesis standards and provide structured judgment regarding confidence in pooled estimates.	The framework was applied consistently across outcomes. It does not modify eligibility or pooled estimates. It enhances transparency regarding confidence in the evidence.
