# Supplementary material for: Physical Exercise After Fragility Fractures: A Systematic Review and Meta-Analysis of Function and Morbidity
Source: J Clin Med. 2026 Apr 10;15(8):2884. doi: 10.3390/jcm15082884 (PMC13116814; doi:10.3390/jcm15082884)
Supplement: Supplementary file 1 [file jcm-15-02884-s001.zip › Table S3. Search strategy per database.pdf]

| Database | Search strategy |
|---|---|
| MEDLINE | (exp Exercise/ OR "Physical activity") AND exp "fractures, bone"/ AND exp Osteoporosis/ |
| PubMed | (Exercise[Mesh] OR "Physical activity") AND "fractures, bone"[Mesh] AND Osteoporosis[Mesh] |
| EMBASE | (exp Exercise/ OR "Physical activity") AND exp "fractures, bone"/ AND exp Osteoporosis/ |
| The Cochrane Library | ([mh Exercise] OR "Physical activity") AND [mh "fractures, bone"] AND [mh Osteoporosis] |
| CINAHL | (MH Exercise+) OR "Physical activity") AND (MH "fractures, bone+") AND (MH Osteoporosis+) |
| WOS | (ALL=Exercise OR "Physical activity") AND ALL="fractures, bone" AND ALL=Osteoporosis |

Table S3.1. Search strategy per database

| Controlled search terms | Free text terms |
|---|---|
| Exercise<br>Fractures, bone<br>Osteoporosis | Physical activity |
| Boolean Operators | |
| “AND”<br>“OR” | |

Table S3.2. Search strategy terms with Boolean operators
