# Supplementary material for: Physical Exercise After Fragility Fractures: A Systematic Review and Meta-Analysis of Function and Morbidity
Source: J Clin Med. 2026 Apr 10;15(8):2884. doi: 10.3390/jcm15082884 (PMC13116814; doi:10.3390/jcm15082884)
Supplement: Supplementary file 1 [file jcm-15-02884-s001.zip › Table S4. List of full-read articles excluded with reason for exclusion and DOI codearticle title for identification.pdf]

Author/year	DOI/PMID	Reason for exclusion
Miko et al., 2018	10.2340/16501977-2349	Duplicated samples
Winther et al., 2020	10.1097/PHM.0000000000001382	No fragility fractures present
Zavala-González et al., 2022	10.1177/02692155221080546	No fragility fractures present
Meng et al., 2022	10.1186/s13018-022-03116-2	No fragility fractures present
Chan et al., 2018	10.1016/j.jfma.2017.05.004	No fragility fractures present
Judd et al., 2024	10.1093/ptj/pzad168	No fragility fractures present
Du et al., 2023	Altern Ther Health Med. 2023;29(8):618–623. DOI not available	No fragility fractures present
Mikkelsen et al.,2019	*Acta Orthop Belg. 85(3):338–345.* **DOI** Not available	No fragility fractures present
Fascio et al., 2022	10.3390/jcm11071766	No fragility fractures present
Nguyen et al., 1998	10.1359/jbmr.1998.13.9.1458	No fragility fractures present
Holsgaard-Larsen et al., 2020	10.1016/j.joca.2020.04.010	No fragility fractures present
Dudoniene et al., 2023	10.3233/BMR-220340	No fragility fractures present
Kemmler et al., 2015	10.1007/s00198-015-3165-3	No fragility fractures present
Tsang et al., 2013	10.1155/2013/827392	No fragility fractures present
Asmidawati et al., 2014	10.1186/1471-2318-14-100	No fragility fractures present
Gram et al., 2014	10.1177/1403494813504505	No fragility fractures present
Drieling et al., 2011	10.1089/jwh.2010.2515	No fragility fractures present
Hendrickx et al., 2014	10.1186/1745-6215-15-392	No fragility fractures present
Beneck et al., 2014	10.1177/0269215514525059	No fragility fractures present
Bailey & Brooke-Wavell, 2010	10.1016/j.bone.2009.12.001	No fragility fractures present
Stolzenberg et al., 2013	10.1055/s-0033-1334870	No fragility fractures present
Gianoudis et al., 2014	10.1002/jbmr.2014	No fragility fractures present
Bennell et al., 2010	doi:10.1186/1471-2474-11-36	No fragility fractures present
Allison et al., 2015	10.1002/jbmr.2499	No fragility fractures present
Tsang et al., 2023	10.1155/2013/827392	No fragility fractures present
Alp et al, 2009	Turkiye Klinikleri J Med Sci. 2009;29(3):687–695. **DOI** Not available	No fragility fractures present
Rackow et al., 2014	10.1111/aphw.12029	No fragility fractures present
Sousa et al., 2014	10.1055/s-0034-1374639	No fragility fractures present
Maciaszek et al., 2007	10.1142/S0192415X07004564	No fragility fractures present
Palombaro et al., 2013	10.2522/ptj.20110476	No fragility fractures present
Madureira et al., 2010	10.1016/j.maturitas.2010.03.009	No fragility fractures present
Toyin Babatunde, 2015	10.1353/hpu.2015.0116	No fragility fractures present
Salis, 2013	10.2466/29.22.PMS.116.3.918-928	No fragility fractures present
Kenny et al., 2010	10.1111/j.1532-5415.2010.03019.x	No fragility fractures present
MacIaszek & Osinski, 2012	10.1142/S0192415X1250019X	No fragility fractures present
Tolomio et al., 2010	10.1080/08952841.2010.518866	No fragility fractures present
Arnold et al., 2008	10.3138/physio.60.4.296	No fragility fractures present
Woo et al., 2007	10.1093/ageing/afm005	No fragility fractures present
Tüzün et al., 2010	Eur J Phys Rehabil Med. 2010;46:69–72. DOI Not available	No fragility fractures present
Trombetti et al., 2011	10.1001/archinternmed.2010.446	No fragility fractures present
Klentrou et al., 2007	10.1123/japa.15.3.287	No fragility fractures present
Madureira et al., 2007	10.1007/s00198-006-0252-5	No fragility fractures present
**Author/year**	**DOI/PMID**	**Reason for exclusion**
Teixeira et al., 2010	10.1007/s00198-009-1002-2	No fragility fractures present

Hourigan et al., 2008	10.1007/s00198-007-0541-7	No fragility fractures present
Rithharomya et al., 2025	10.60099/prijnr.2025.269063	No fragility fractures present
Williams et al., 2025	10.1136/bmjopen-2024-091603	No fragility fractures present
Xinhan et al., 2025	10.62177/apjcmr.v1i1.239	No fragility fractures present
Østergaard et al., 2024	10.1016/j.jse.2023.12.002	No fragility fractures present
Prieto-Moreno et al., 2025	10.1016/j.eclinm.2024.102677	No fragility fractures present
Yaacobi et al., 2025	10.1007/s41999-025-01192-1	No fragility fractures present
Suikkanen et al., 2026	10.1177/02692155251389435	No fragility fractures present
Kistler-Fischbacher et al., 2025	10.1093/jbmr/zjaf058	No fragility fractures present
Zeng et al., 2025	10.1093/ageing/afaf349	No fragility fractures present
Sabino-Serra et al., 2024	10.1016/j.jbmt.2023.09.004	No fragility fractures present
Ji et al., 2025	10.1001/jamanetworkopen.2025.43278	No fragility fractures present
Heo et al., 2024	10.1016/j.archger.2023.105136	No fragility fractures present
George et al., 2025	10.1016/j.jbmt.2025.10.033	No fragility fractures present
Falck et al., 2024	10.1007/s40520-024-02766-y	No fragility fractures present
Williams et al., 2025	10.3310/RBGD4741	No fragility fractures present
Prescott et al., 2026	10.3310/GJAC1602	No fragility fractures present
Tuan et al., 2024	10.2196/59468	No fragility fractures present
Hall et al., 2025	10.3310/GJAC2501	No fragility fractures present
Subías-Perié et al., 2025	10.1016/j.exger.2025.112911	No fragility fractures present
Kim et al., 2025	10.1186/s12877-025-06679-x	No fragility fractures present
Killingmo et al., 2024	10.1136/bmjopen-2023-079704	No fragility fractures present
Sørensen et al., 2025	10.1111/sms.70154	No fragility fractures present
Traistary et al., 2020	10.1007/s00198-020-05696-3	Study yields no results (congress communication)
Ho et al., 2019	10.1007/s00198-019-04993-w	Study yields no results (congress communication)
Bu & Wang, 2019	10.1111/bcpt.13494	Study yields no results (congress communication)
Hadamus et al., 2019	10.1093/ageing/afz164.130	Study yields no results
Prieto-Moreno et al., 2023	10.1136/annrheumdis-2023-eular.5548	Study yields no results
Giangregorio et al., 2018	10.1007/s00198-018-4652-0	Study yields no results
Dwivedi, 2020	10.1177/1759720X20969289	Study yields no results
Mora-Traverso et al., 2022	10.1002/nur.22218	Study yields no results
Yashkov et al., 2021	10.17116/kurort20219806231	Study yields no results
Che et al., 2023	10.1186/s12891-023-06806-y	Type of study
Drăgoi et al., 2010	Rom J Morphol Embryol. 51(4):707–711. DOI Not available	Type of study
Thomas et al., 2008	10.1186/1471-2318-8-4	Type of study
Cook et al., 2011	10.1186/1471-2318-11-30	Type of study
McArthur et al., 2021	10.1080/09638288.2019.1696418	Type of study
Dimitriou et al., 2012	10.2217/ijr.11.68	Type of study
Selecki & Eisman, 2014	Medicine Today. 2014;15(11):18–27. DOI Not available	Type of study
Giangregorio et al., 2014	10.2522/ptj.20130625	Type of study
Marcinkowska et al., 2013	Endokrynol Pol. 2013;64(2):108–113. PMID: 23653273	Type of study
Mutchie et al., 2022	10.1519/JPT.0000000000000310	Type of study
Van Ooijen et al., 2013	10.1186/1471-2318-13-34	Type of study

Gaboury et al., 2013	10.1186/1748-5908-8-10	Type of study
Barker et al., 2014	10.1186/1745-6215-15-22	Type of study
Sheehan & Bastas, 2024	10.1186/ISRCTN16147125	Type of study
Pulkkinen et al., 2011	10.1016/j.bone.2011.07.022	Type of study
Longo et al., 2012	10.1093/bmb/ldr048	Type of study
Barcelos et al., 2024	10.1007/s00198-024-07265-4	Type of study
Çergel et al., 2019	10.1007/s11657-019-0632-z	Type of activity program
Masiero et al., 2020	10.1007/s00484-019-01846-3	Type of activity program
Jinli-Guo et al., 2019	10.1186/s13018-019-1295-6	Type of activity program
Said et al., 2021	10.1186/s12877-021-02321-8	Type of activity program
Helynen et al., 2023	10.1007/s00402-022-04406-4	Type of activity program
Reh et al., 2019	10.3390/brainsci9030066	Type of activity program
Ebrahim et al., 1997	10.1093/ageing/26.4.253	Type of activity program
Davis et al., 2007	10.1093/gerona/62.8.888	Type of activity program
Beckmann et al., 2021	10.1002/pri.1896	Type of activity program
Liu et al., 2021	PMCID: PMC8205694	Type of activity program
Zusman et al., 2019	10.1519/JPT.0000000000000193	Type of activity program
Terzis et al., 2021	10.22540/jfsf-06-057	Type of activity program
Cheng et al., 2022	10.1142/S101370252250010X	Type of activity program
Lim et al., 2025	10.1016/j.jamda.2024.105321	Type of activity program
**Author/year**	**DOI/PMID**	**Reason for exclusion**
Karakasidou et al., 2013	Motor control exercise can reduce pain and improve postural alignment in osteoporotic women with vertebral fractures: a randomized controlled trial. DOI Not available	Type of activity program
Monaghan et al., 2012	10.1186/1471-2474-13-237	Type of activity program
Merle et al., 2017	10.1007/s00198-017-3953-z	Type of activity program
Howell et al., 2023	10.3390/ijerph20043107	Type of activity program
Corna et al., 2021	10.1177/0269215520968694	Type of activity program
Woodward et al., 2014	10.1186/1471-2318-14-140	Type of activity program

Table S4. List of full-read articles excluded with reason for exclusion and DOI code/article title for identification.
