# Supplementary material for: Physical Exercise After Fragility Fractures: A Systematic Review and Meta-Analysis of Function and Morbidity
Source: J Clin Med. 2026 Apr 10;15(8):2884. doi: 10.3390/jcm15082884 (PMC13116814; doi:10.3390/jcm15082884)
Supplement: Supplementary file 1 [file jcm-15-02884-s001.zip › Table S5. GRADE rating.pdf]

| Certainty assessment | | | | | | | Summary of findings | | | | |
|---|---|---|---|---|---|---|---|---|---|---|---|
| | | | | | | | Study event rates (%) | | | Anticipated absolute effects | |
| Participants (studies) Follow-up | Risk of bias | Inconsistency | Indirectness | Imprecision | Publication bias | Overall certainty of evidence | With | With structured exercise programs | Relative effect (95% CI) | Risk with | Risk difference with structured exercise programs |
| **Time Up and Go (follow-up: range 3 months to 3 months; assessed with: seconds)** | | | | | | | | | | | |
| 532 (3 RCTs) | serious[a] | serious[b] | not serious | serious[c] | all plausible residual confounding would reduce the demonstrated effect dose response gradient | ⊕⊕⊕◯ Moderate[a,b,c] | 266 | 266 | - | 266 | MD **3.27 lower** (7.59 lower to 1.04 higher) |
| **Short Physical Performance Battery (follow-up: range 12 months to 12 months; assessed with: points)** | | | | | | | | | | | |
| 578 (4 RCTs) | very serious[a,d] | serious[e] | not serious | serious[c] | all plausible residual confounding would reduce the demonstrated effect dose response gradient | ⊕⊕◯◯ Low[a,c,d,e] | 296 | 282 | - | 296 | MD **1.01 higher** (0.11 higher to 1.91 higher) |

| Certainty assessment | | | | | | | Summary of findings | | | | |
|---|---|---|---|---|---|---|---|---|---|---|---|
| **QUALEFFO-41 pain (follow-up: range 12 months to 12 months; assessed with: points)** | | | | | | | | | | | |
| 515 (4 RCTs) | very serious[a,d,f,g] | serious[h] | not serious | not serious | all plausible residual confounding would reduce the demonstrated effect dose response gradient | ⨁⨁⨁◯ Moderate[a,d,f,g,h] | 248 | 267 | - | 248 | MD **11.61 lower** (22.99 lower to 0.23 lower) |

**CI:** confidence interval; **MD:** mean difference

**Explanations**

a. No blind personnel and participants to which group they were assigned

b. Substantial heterogeneity ($I^2 = 94\%$). Sensitivity analyses showed the pooled effect estimate was unstable, with significance and effect size changing when individual studies were excluded, indicating inconsistent results.

c. The confidence interval crosses no effect line, including a significant benefit and no effect, indicating imprecision.

d. There is some risk of bias from incomplete data; 63 of 615 (10%)7 (3%) were non-contactable at 4 months, rising to 23 (4%) at 12 months. Loss was highest in the exercise arm but less than 10% overall.

e. Moderate heterogeneity ($I^2 = 64\%$). Sensitivity analyses showed the pooled effect was unstable, with significance depending on excluding specific studies.

f. Not reported random sequence generation in one study

g. Not reported allocation concealtment in one study

h. Substantial heterogeneity ($I^2 = 73\%$). Sensitivity analyses revealed unstable pooled effects, with the effect's direction and significance depending on individual study inclusion studies.
